# Therapeutical and Pharmaceutical Notes

**Published:** 1901-07

**Authors:** W. J. Martin

**Affiliations:** Kankakee, Ill.


### THERAPEUTICAL AND PHARMACEUTICAL NOTES.

UNDER THE DIRECTION OF W. J. MARTIN, M.D.C.,
KANKAKEE, ILL.

---

DAIRY BACTERIA. The progress which has been made in recent years in determining the useful and injurious dairy bacteria and the means of controlling their growth has greatly promoted the intelligent production and handling of milk for household consumption and in butter-making. In this work a number of the agricultural experiment stations have taken an important part. The Storrs Experiment Station in Connecticut is among this number, and its twelfth annual report, just issued, gives an interesting résumé of something over two hundred types of bacteria which Professor Conn has found in dairy products during the ten years he has been engaged in this work. On the basis of his studies he proposes a classification of dairy bacteria. Although the total number of species found in dairy products is large, only a comparatively few occur with very great regularity. Professor Conn concludes that those of the region represented by his investigations consist chiefly of three groups of closely related bacteria. Of these the most abundant are bacteria acidi lactici I. (Esten) and B. acidi lactici II. (new species), which constitute the first group. The former occurs almost universally in milk and cream, is nearly always present in sour milk, and has been found by far the most abundant in all samples of ripened cream examined. The second form, while very abundant in sour milk and cream, occurs in less numbers. Several of the poor commercial cultures for ripening cream in butter-making consist of bacteria of this type. The next most important group is represented by a species regarded as identical with B. lactis aërogenes, and includes a number of types of great similarity but with different physiological characters. It has been found almost universally in milk, but never in very great numbers. Some of the pure cultures used in Europe for cream ripening appear to belong to this group. Typical sour milk, with its tendency to fragmentation and its sour odor, Professor Conn thinks, is never developed without the aid of some of the organisms of this group. The third type is the micrococcus lactis variens of the author. It is common in fresh milk, and is thought to exist in the milk ducts, which is not the case with the preceding types, the source of contamination with which is believed to be entirely external. It is commonly overgrown by the lactic organisms, and is less common in old milk. While the classification of dairy bacteria is regarded as necessarily a tentative one, it is offered as a basis for bringing together the work of American dairy bacteriologists.

Another paper in this report bearing on the subject of dairying relates to the use of milk of tuberculous cows—a matter of more than usual interest in view of the attention which is being given to the general subject of tuberculosis and its transmission. Experiments in using the milk of tuberculous cows for feeding calves at the Storrs station have been in progress for several years. During the first two years, when the cows had the disease only in its earliest stages, the young cattle which received their milk and ran with them constantly exhibited no signs of the disease as far as could be detected by the tuberculin-test or physical examination; but the result for the next year and a half was quite different. Five calves were fed the milk of these same cows, and all five responded to the tuberculin-test and proved to be diseased. The physical condition of three of the cows indicated that during the last year the disease had progressed decidedly in them. While the results indicate that the danger from the spread of tuberculosis to other animals through the milk is not always as great as has been supposed, they suggest the exercise of greater caution in excluding from use for supplying faulty milk all cows in which the disease is sufficiently advanced to be detected. Experiments at a number of places have shown that the milk of tuberculous cows may be pasteurized and safely used for raising calves, but precautions should be taken to insure confining its use to this purpose.—*Popular Science Monthly*.

VERATRUM VIRIDE. A. B. Isham (*Medical News*) explains the *modus operandi* of this drug by the excessive glandular activity which it excites. Not only are the sweat glands stimulated, but the salivary glands pour out an abundant secretion and the biliary flow is greatly increased. The cells of the liver are undoubtedly excited to greatly increased activity through a direct action of the drug upon them. The excitation of the salivary and sweat glands has been ascribed by investigators to vasomotor paralysis and consequent decrease in pressure in the peripheral bloodvessels, but there is never noted such an outpouring of fluids in cases of spinal injury with apparently complete vasomotor paralysis. In addition to this vasomotor influence there must be some excitory action of the drug upon the gland-cell. The removal of waste elements through the transudation of the skin may reduce the fever by checking metabolism, by evaporation from the surface, or by retraction of the neurons consequent upon the abstraction of fluid from the tissues, thereby cutting off the passage of nervous irritation. This last theory is accepted by the author as better than the statement that the drug is a heart depressant. A similar retraction or contraction, he thinks, takes place in the heart muscles. Phagocytosis continues energetically.

FORMALIN IN GLYCERIN. A. C. Jordan (*Lancet*) has found that by combining formalin with glycerin the irritating nature of the drug and the pain which it causes may be prevented. He uses a mixture of 1 to 4 per cent. It is best prepared fresh. Applied to the throat with a brush, a single application can be depended upon to kill every microörganism with which it comes in contact. In follicular tonsillitis formalin in glycerin (2 to 4 per cent.) is a specific if used before there is deep collection of pus. After a single thorough application the temperature falls to normal within a few hours and remains normal. The application is usually attended by a little soreness, lasting only a few hours. Formalin in glycerin is useful in all parasitic skin diseases, especially in tinea tonsurans. The whole area is cleansed by soft soap and water, and a 4 per cent. solution of formalin in glycerin is applied.

TRANSPLANTATION OF A PIECE OF COLON INTO THE STOMACH. Reerink (*Ziegler's Beit. f. Allg. Pathologie*) performed the experiment on a number of dogs of transplanting a piece of colon, connected with its mesocolon, into the anterior wall of the stomach. An autopsy was performed on one of the dogs four weeks after the operation, and Reerink found the transplanted gut had not only healed perfectly, but was continuing its functions. Experiments in which no pedicle was left to the flap invariably failed; the omentum was adherent to the stomach wall, and the portion of the bowel was shrivelled up, and in some instances had disappeared entirely by absorption. The author demonstrated that a large part of the anterior stomach wall can easily be substituted, and that the transverse colon, because of its size, is especially adapted for this transplantation. A further report of the dogs now under observation is promised at a later time.

Neurotomy in Cases of Hock Lameness. Veterinary Surgeon Overbeck reports that, having heard of so many favorable results following section of the tibial and the peroneal nerves in such cases, he determined to try the treatment on a five-year-old carriage mare. The animal was very lame on account of extensive congestion over all the internal aspect of the hock ; very frequently when leaving the stable she could hardly walk, and supported herself on the point of her toes. Three weeks after cutting of the tibial nerve the wound caused by the operation had become cicatrized and the lameness had greatly diminished. The external aspect was then operated on, and three weeks later a distinct improvement was observable, but the lameness had not entirely disappeared. The mare was put into harness a fortnight later, and went quite easy after a quarter of an hour's work. Next day, however, a slight lameness was observable, and at the same time a slight congestion on the inner aspect of the hock. She was put again into harness, but at the end of half an hour she stopped suddenly and showed a fracture of the pastern of the same leg. She was ordered to be slaughtered, and the post-mortem showed a multiple fracture of the first phalanx. In a second case—that of a seven-year-old mare—very lame on the off hind leg, in consequence of an enormous spavin, the same operation was performed, and at the end of five weeks the lameness had entirely disappeared; but, unfortunately, after the animal had been twice in harness, she showed a very pronounced congestion of the whole of the hock, and the lameness returned with all its old intensity. The owner sold the mare, and M. Overbeck lost trace of her; but he thinks the results must have been unfavorable. He is of the opinion that the operation is far from harmless, and he bases this opinion largely on the serious arthritis which showed itself in the second case. He thinks recourse should be had to the operation only as a last resort.—(*Tijdschrift voor veeartsenijkunde*) *Veterinary Journal.*

Persistence of Microbes in Milk of Cows which have Suffered from Mammitis. An epidemic of diarrhœic enteritis broke out in a household after using boiled milk. Drs. Lameris and Van Harrevelt examined the stable from which the milk had come, and found there several cows which had recently recovered from mammitis. One animal was still suffering from this disease, which was located in one of the quarters. This latter gave only an abnormal product of secretion, which was collected antiseptically as well as the milk from the cows which had been formerly affected and were now cured. Cultivations were begun, partly with samples previously subjected to boiling, partly with others just as they were collected. The latter alone "grew." There was thus obtained as pure culture a streptococcus, aërobic and anaërobic, easily colorable, non-pathogenic either for rabbits or guinea-pigs. The two doctors think there is no doubt that this microbe is the efficient cause of the mammitis, and it is really curious to notice that it was found in such abundance after several days in the apparently normal milk of cows completely cured. Boiling having destroyed the vitality of the streptococci, the diarrhœa must have been caused by toxins which had resisted the boiling. We may, therefore, conclude that milk from cows which have suffered from mammitis may retain its hurtfulness for at least eight days after cure.—*Zeitschrift f. Milchhygiene.*

